# Three cycles of AC chemotherapy regimen increased oxidative stress in breast cancer patients: A clinical hint

**DOI:** 10.22088/cjim.8.4.264

**Published:** 2017

**Authors:** Mostafa Taherkhani, Soleiman Mahjoub, Dariush Moslemi, Ahmad Karkhah

**Affiliations:** 1 Student Research Committee, Babol University of Medical Sciences, Babol, Iran.; 2 Cellular and Molecular Biology Research Center, Health Research Institute, Babol University of Medical Sciences, Babol, Iran.; 3 Department of Clinical Biochemistry, School of Medicine, Babol University of Medical Sciences, Babol, Iran.; 4 Cancer Research Center, Health Research Institute, Babol University of Medical Sciences, Babol, Iran.

**Keywords:** Breast cancer, Chemotherapy, Malondialdehyde, Oxidative stress, Total antioxidant status

## Abstract

**Background:**

Recent studies have suggested the importance of oxidant/antioxidant status in initiation and progression of breast cancer. The aim of this study was to evaluate oxidative stress markers in breast cancer patients before and after 3 cycles of chemotherapy with adriamycin and cytoxan (AC). Also, in this study the effect of age and the stage of disease on oxidative stress markers were compared and evaluated.

**Methods:**

This study included 60 women with newly diagnosed stage II-III breast cancer who underwent chemotherapy with AC as the therapy-first strategy after surgery. Serum samples were obtained before treatment and after the third chemotherapy. Then, serum total antioxidant status (TAS) and malondialdehyde (MDA) as lipid peroxidation marker were analyzed. Moreover, the effects of the subject’s age and clinical disease stage were investigated.

**Results:**

A concurrent significant increase in MDA (p<0.001) and a significant decrease in TAS (p<0.001) were also observed after 3 cycles of AC chemotherapy. In addition, some changes were found in the status of oxidative stress markers which were associated with age and clinical disease stage.

**Conclusion:**

Our data indicated that chemotherapy with AC increase the oxidative stress in breast cancer patients. The present study indicated that higher stages of the breast cancer are associated with significant increases of oxidative stress markers.

Breast cancer is the most common cancer among women and also the second leading cause of death worldwide (1). The incidence of breast cancer has increased in Iranian population due to numerous predisposing factors in particular lifestyle (2). The high incidence rate of breast cancer has attracted many researchers to introduce new therapeutic approaches or improve pervious strategies. Several studies suggest that oxidative stress is implicated in etiology and progression of breast cancer. Oxidative stress is usually defined as a state in which the balance between production of the reactive oxygen species (ROS) and the efficiency of the antioxidant defense is disturbed. On the other hand, oxidative stress leads to a significant production of ROS which overcomes the antioxidant defense system or when there is a major decrease in antioxidant defense (3, 4). Various cellular defense mechanisms consisting of enzymatic components such as catalase, glutathione peroxidase, superoxide dismutase and non-enzymatic components such as vitamin E, vitamin C, and glutathione components control the standard oxidant-antioxidant balance in the body (5). Excessive produced ROS could alter and damage many intracellular components including lipids, proteins and nucleic acids (4). Furthermore, reactive species also lead to formation of nicks in the structure of DNA and malfunctions in the DNA repair mechanism where inactivation or loss of certain tumor suppressor genes might appear (6). 

Recently many reports have also indicated that the antineoplastic agents including 5-fluorouracil, doxorubicin and cyclophosphamide used extensively for treatment of cancer lead to a considerable reduction in antioxidant levels (7). Cellular toxicity of these antineoplastic drugs enhances the peroxidation of the unsaturated fatty acids of membrane phospholipids which increase the permeability of the cell membrane and could lead to cell death (8).

Because of high incidence of the disease in Iranian women as well as the importance of oxidation by these anticancer drugs, this current study was conducted to investigate the oxidative stress markers in breast cancer patients and the impact of age and clinical stage on these variables before and after three AC chemotherapy cycles.

## Methods

**Patients and study design: **This study included 60 breast cancer patients from Shahid Rajaee Hospital in Babolsar, Iran. Tumors were classified by the Pathological tumor-node-metastasis (pTNM) staging system in accordance with the diagnostic criteria of the American Joint Committee on Cancer Classification System. Our study was approved by the Ethics Committee of Babol University of Medical Sciences and all patients signed a term of free informed consent. The clinical criteria for this study included age at diagnosis, tumor side, clinical stage and histologic grade presented in table 1. Furthermore, none of the patients received previous therapy including immunomodulators, cytokines or steroids. Blood samples were collected from patients before undergoing any type of treatment and after receiving three cycles of AC chemotherapy. Chemotherapy regimen for 6 cycles was AC-T (adriamycin 60 mg/m2, cytoxan 600 mg/m2, taxotere 80 mg/m2).

**Sample collection and preparation: **Blood samples were collected from patients by arterial puncture arm before undergoing any type of treatment and after combination chemotherapy. Sera was separated following centrifugation at 3,000 g for 15 minutes and immediately stored at –80^o^ C until biochemical analysis**.**

**Biochemical analysis**

**Evaluation of serum malondialdehyde: **The level of thiobarbituric acid reactive substances (TBRAS) was measured as an index of lipid peroxidation. Malondialdehyde (MDA) is one of the most important TBARS compounds (9). The working TBARS solution contained 0.375% TBA and 15% TCA (Trichloroacetic acid, Sigma) in 0.25 N HCl; TCA-TBA-HCl reagent was freshly prepared and 2 ml of this solution was mixed with 0.5 ml serum or standard in a test tube. The solution was heated for 30 min in a boiling water bath. After cooling, the absorbance was measured at 532 nm against a blank that contains all the reagents minus serum. The standard curve was prepared using serial concentrations of 1,1,3,3-tetraethoxypropane (Sigma, St. Louis, MO, USA). The malondialdehyde thiobarbituric acid (MDA-TBA) adduct was shown at 532 nm and quantified by reference to a standard curve of 1,1,3,3-tetraethoxypropane, submitted to the TBA colorimetric procedure (10).

**Assay of total antioxidant status (TAS): **Total antioxidant status was determined by the ferric reducing antioxidant power (FRAP) assay. The FRAP reagent was prepared from 10 mmol/L TPTZ solution in HCl 40 mmol/L plus FeCl3 (20 mmol/L) and acetate buffer (0.3 mol/L, pH 3.6) in a 1:1:10 ratio. Freshly prepared FRAP reagent was warmed at 37^o^C for 10 min. Serum sample or standard (50 μL) was mixed with 1.5 ml of FRAP reagent in a test tube and incubated at 37^o^C for 10 min. Then, the absorbance of the ferrous tripyridyl triazine complex as colored product was measured at 593 nm and compared to the blank. The FRAP standard curve was obtained from absorbance values of iron sulfate as standard with serial concentrations (125, 250, 500, 1000 μM) (11).

**Statistical analysis: **The analysis was performed using SPSS Version 18.0 software. Differences between groups for comparing pre-and post-chemotherapy data by paired t-test and between oxidative stress markers in each group were determined by independent t-test analysis. A p<0.05 was defined statistically significant.

## Results

**Figure 1:**
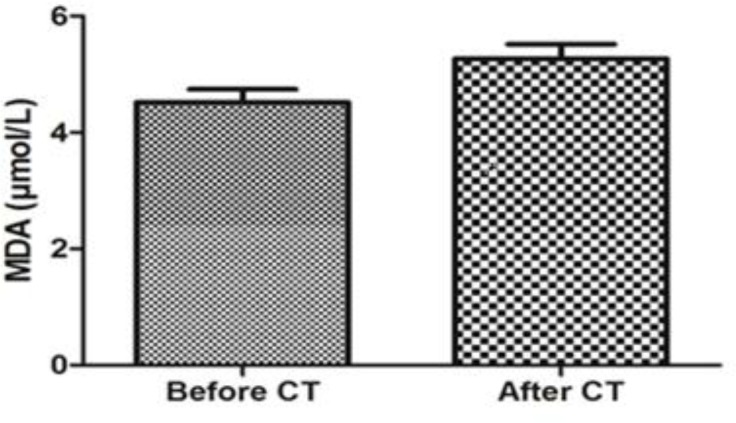
Serum Malondialdehyde (MDA) levels before and after chemotherapy in breast cancer patients

**Figure 2:**
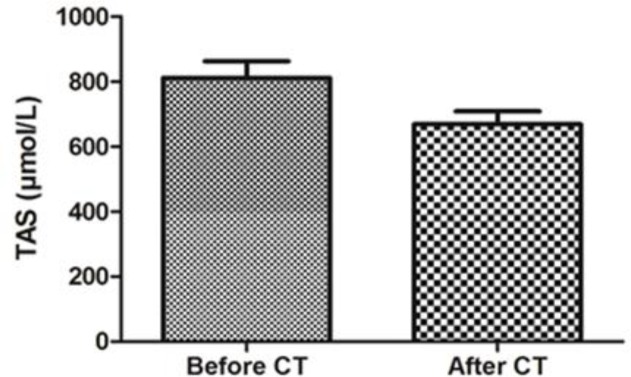
. Serum total antioxidant status (TAS) levels before and after chemotherapy in breast cancer patients

Table 1 shows the demographic and clinical data of breast cancer patient participants in our study. The median age of the patients was 48 (31–62) years. As it is obvious in this table, patients were in stages II and III of the disease. Obtained results showed that MDA level increased significantly in breast cancer patients after combination chemotherapy with AC (p<0.05). Therefore, AC chemotherapy triggered an enhancement in the lipid peroxidation compared to early status (fig 1). In addition, breast cancer patients treated with AC also showed significantly lower total antioxidant status (TAS) in comparison with before combination chemotherapy (fig 2).

**Table 1:** Characteristics of 60 patients with breast cancer

**Characteristics**	**No. of patients**
**Age, years (median, range**)	48 (31-62)
**Tumor side**	
Left breast Right breast	31 29
**Histological grade**	
Grade I, II Grade III	47 13
**Stage status**	
Stage II Stage III	36 24

All of the analyzed variables were categorized in two age groups (≤ 48 and >48 years old) and the clinical stages of the diseases were II and III. Between two age groups, there were no significant differences in MDA and TAS before and after the three cycles chemotherapy (table 2). In two age groups, chemotherapy led to a significant decrease in TAS and increase MDA levels.

**Table 2:** Serum levels of oxidative stress markers before and after chemotherapy in different age groups

**Age (years)**	**≤48** **(n=34)**	**>48** **(n=26)**	**P value**
**Total antioxidant status (TAS)**			
Before After P value	831±190 670±156 < 0.001	786±212 665±157 < 0.001	0.289 0.801
**Malondialdehyde (MDA)**			
Before After P value	4.3±0.5 5.2±0.8 < 0.001	4.6±1.1 5.3±1.0 < 0.001	0.186 0.881

The effects of three cycles chemotherapy using AC on the measured variables were compared in these two stages of disease before and after chemotherapy. Our finding demonstrated that TAS and MDA levels showed statistically significant difference in comparison with pretreatment levels in the two groups according to clinical stages (table 3) and tumor grades of breast cancer (table 4).

**Table 3:** Serum levels of oxidant/antioxidant status before and after chemotherapy in different clinical stages of breast cancer

**Variables**		**stage II ** **(n=36)**	**stage III** ** (n=24)**	**P value**
TAS	Before	844±179	760±221	0.112
	After	713±138	600±157	0.005
	P value	< 0.001	< 0.001	
MDA	Before	4.27±0.571	4.88±1.08	0.007
	After	4.92±0.779	5.74±0.94	0.001
	P value	< 0.001	< 0.001	

**Table 4:** Serum levels of oxidant/antioxidant status before and after chemotherapy according to tumor grades

**Variables**		**Grade I and II** **(n=47)**	**Grade III** **(n=13)**	**P value**
TAS	Before	823±170	768±287	0.388
	After	681±146	622±184	0.233
	P value	< 0.001	0.027	
MDA	Before	4.34±0.582	5.13±1.35	0.003
	After	5.11±0.831	5.81±1.19	0.019
	P value	< 0.001	0.002	

## Discussion

Several investigations have indicated that ROS are involved in the etiology and progression of breast cancer. Elevated rates of reactive oxygen species (ROS) have been detected in almost all cancers, where they promote many aspects of tumor development and progression. On the other hand, a delicate balance of intracellular ROS levels is required for cancer cell function (4, 12). Elevated ROS levels can initiate DNA damage, and might ultimately lead to carcinogenesis (6). There are many studies based on breast cancer culture cells or experimental data, whereas few studies have investigated the changes of oxidative stress parameters in patients with breast cancer after combination chemotherapy with a specific regimen. Thus, this study was conducted to investigate the effect of combination chemotherapy with Adriamycin and Cytoxan (AC) on the levels of oxidative stress markers.

In the current study, our observations indicated that oxidative stress markers increased in breast cancer patients after 3 cycles of chemotherapy with AC. On the other hand, we found that combination chemotherapy using AC increased TBARS, and decreased TAS. These findings are compatible with previous reports on enhanced generation of lipid peroxidation products in response to chemotherapy. Nowak et al. demonstrated that chemotherapy composed of carboplatin, etoposide and vincristine increases the serum levels of end-products of lipid peroxidation in a majority of Small Cell Lung Cancer (SCLC) patients (13). In another study done by Look and Musch, repetitive polychemotherapy with radical-generating compounds resulted in a significant decrease in antioxidant capacity in cancer patients and hence led to oxidative stress (14). Additionally, in patients with higher stage and grade we observed more increase in induction of oxidative stress (tables 3, 4). High levels of TBARS and decreased TAS in chemotherapy may result in high cytotoxic activity in breast cancer patients.

Consequently, regulation of the MDA and TAS production is important for the improvement of breast cancer. When we compared our results in different stages such as II and III, we found that interestingly, there was a significant difference in MDA levels, before and after the chemotherapy between two groups. Besides, these patients had dramatically reduction in the amount of TAS.

Oxidative stress markers in patients younger than 48 years and people older than 48 years were measured before and after chemotherapy. Obtained results revealed that chemotherapy with AC triggered oxidative stress in two age groups. Taken together, our data indicate that AC chemotherapy regimen increases the oxidative stress in breast cancer patients. The limitation of the current study was diet management because this may be difficult or sometimes impossible for the cancer patients.

In conclusion, monitoring of serum oxidative stress markers may be helpful for breast cancer patients in the evaluation of chemotherapy effects. Our data indicated that chemotherapy with AC increased malondialdehyde as a lipid peroxidation marker and decreased total antioxidant status in breast cancer patients. The present study showed that the higher stages of breast cancer are associated with significant increases of oxidative stress markers**.**

Future studies need to investigate the uses of potent antioxidant compounds to compensate the oxidative stress after chemotherapy in breast cancer patients.
